# Recent advances in understanding and managing mitral valve disease

**DOI:** 10.12688/f1000research.16066.1

**Published:** 2019-09-24

**Authors:** Wendy Tsang

**Affiliations:** 1 Toronto General Hospital, University Health Network, Toronto, Ontario, Canada

**Keywords:** Mitral valve, mitral regurgitation, rheumatic mitral stenosis, prosthetic valves, management, transcatheter mitral valve procedures.

## Abstract

Interest in the mitral valve has increased over the past few years with the development of new technologies that allow intervention in patients previously deemed too ill for treatment. This increased attention has resulted in a significant increase in publications on the mitral valve, the majority of which focus on mitral regurgitation and mitral valve surgery/intervention. The focus of this review is on publications in the past few years that offer additional insights into our understanding and management of mitral valve disease and specifically mitral regurgitation. It will discuss mitral valve anatomy, epidemiology of mitral valve disease, changes in the 2017 management guidelines, management of mitral bioprosthetic valves, transcatheter mitral valve procedures and the repair of rheumatic valves.

## Introduction

**Figure 1.**
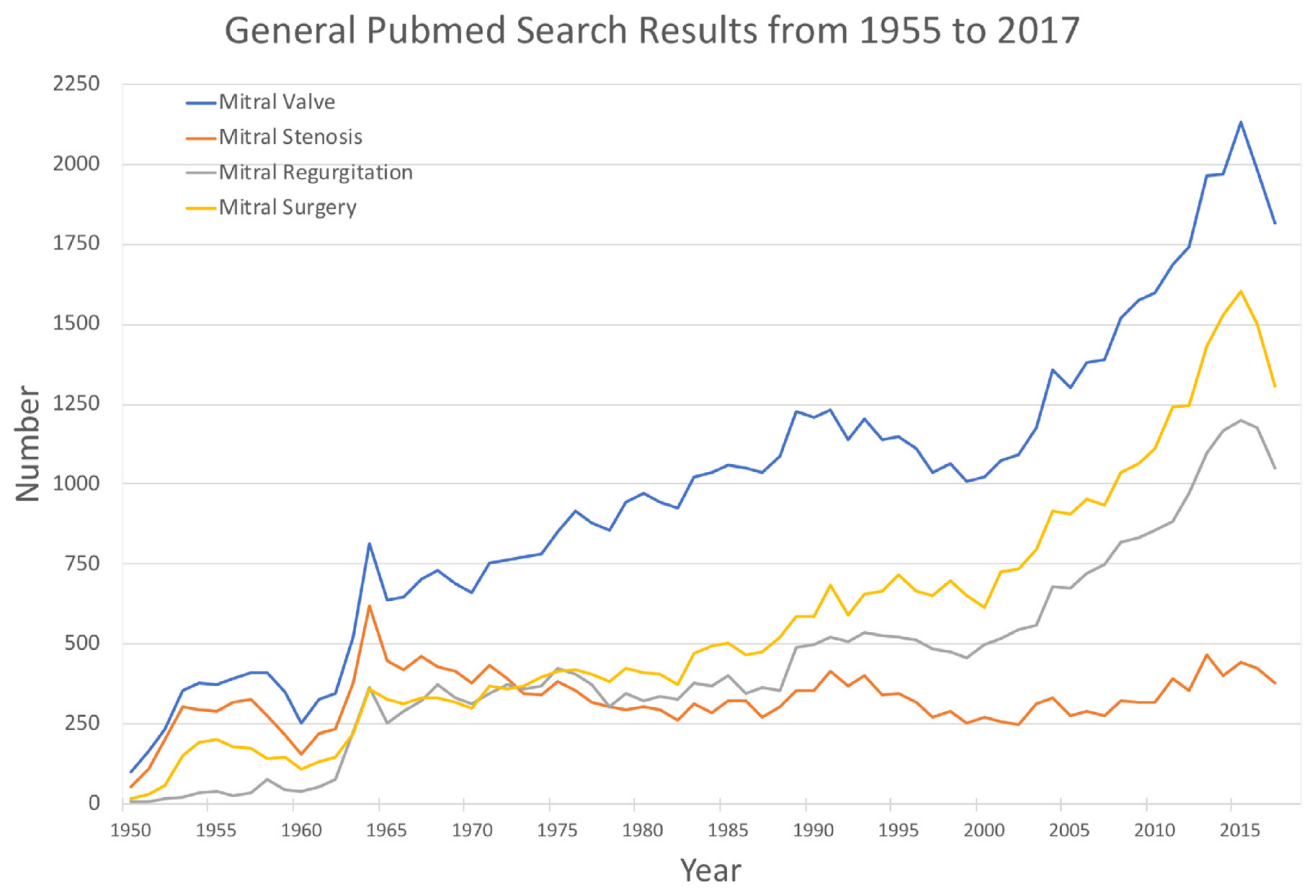
General PubMed search results related to the mitral valve per year from 1955 to 2017.

Interest in the mitral valve has increased over the past few years with the development of new technologies that allow intervention in patients previously deemed too ill for treatment. This increased attention has resulted in a significant increase in publications on the mitral valve, the majority of which focus on mitral regurgitation and mitral valve surgery/intervention ( Figure 1). Additionally, numerous societal guidelines and consensus statements have been published to standardize assessment and management ^1– 6^. The focus of this review is on publications in the past few years that offer additional insights into our understanding and management of mitral valve disease and specifically mitral regurgitation. It will discuss mitral valve anatomy, epidemiology of mitral valve disease, changes in the 2017 management guidelines, management of mitral bioprosthetic valves and transcatheter mitral valve procedures.

## Mitral valve anatomy

Insights into mitral valve anatomy and the changes that occur from intrinsic valvular abnormalities or secondary to other cardiac pathologies continue to evolve. This is due to advancements in cardiac imaging that allows us to visualize and quantify the mitral valve in three dimensions. In particular, there have been gains in understanding the pathomorphological mitral annular and leaflet and left ventricular myocardial changes that occur with myxomatous mitral valve disease. Additionally, there has been greater appreciation of the significance of mitral clefts and cleft-like indentations during percutaneous interventions.

With three-dimensional echocardiography, it is well established that the normal mitral annulus is saddle-shaped and that there are dynamic changes to this shape during the cardiac cycle ^7^. Alterations to mitral annular size and function have been recognized secondary to pathologies such as myxomatous mitral valve disease and ischemic cardiomyopathies ^8, 9^. The mitral annulus of patients with myxomatous mitral valve disease dilates in all directions. Although it retains its dynamic changes during the cardiac cycle, it loses its regurgitation preventive mechanism through decoupling of annular and ventricular contraction ^10^. In contrast, in patients with ischemic cardiomyopathies, the mitral annulus enlarges only in the anteroposterior diameter and is adynamic throughout the cardiac cycle. Developments over the past few years have given us a greater appreciation that mitral annular remodeling differs not only between primary and secondary mitral regurgitation but also between primary etiologies such as Barlow’s disease and fibroelastic deficiency. These pathophysiological differences involve not only mitral annular dynamics but also leaflet tissue changes ( Table 1) ^11, 12^.

**Table 1.** Mitral annular and leaflet changes in mitral valve disease.

Disease	Mitral annulus	Mitral leaflets
Fibroelastic deficiency	• Moderately dilated • Preserved shape • Relatively preserved contraction pattern	• Moderately increased length and area • Smaller prolapse volume compared with patients with Barlow’s disease • Minimal distensibility during systole
Barlow’s disease	• Severely dilated • Flattened shape • Loss of systolic accentuation of the saddle shape • Annular-ventricular decoupling	• Severely increased length and area • Larger prolapse volume compared with patients with fibroelastic deficiency • Significant distensibility during mid and late systole
Ischemic/Dilated cardiomyopathy	• Dilated • Adynamic	• Mild increase in length and area • No prolapse • Tethering

There are primary abnormalities of the mitral annulus that likely contribute to mitral regurgitation severity in patients with Barlow’s disease. Annular dynamics in patients with Barlow’s disease are blunted more than what would be expected from the amount of ventricular and atrial remodeling observed ^11^. This may be related to the mitral annular disjunction detected in patients with myxomatous mitral valve disease ^13^. However, minor degrees of mitral annular disjunction have been measured in “normal” patients ^13, 14^. For these patients, it is unclear whether this represents early pathological changes or is a normal variant. It must be noted that the mitral annulus is histologically a non-continuous structure that varies in form and has different degrees of stiffness depending on the regional dense collagen content ^15^. This has resulted in different anatomic, surgical and echocardiographic definitions ( Table 2) ^15^. The non-continuous form of the annulus may contribute to the changes seen not only in patients with Barlow’s disease but also in normal patients. However, further mechanistic studies are needed to understand these changes.

**Table 2.** Definitions of the mitral annulus.

Mitral annulus	Definition
Anatomic	The fibroelastic structure at the level of the atrioventricular junction that separates the left atrium and ventricle
Surgical	The transition zone seen between the left atrium myocardium and the mitral leaflet
Echocardiographic	The hinge region of the mitral leaflet

Differences in leaflet distensibility or reserve are also observed between patients with Barlow’s disease and those with fibroelastic deficiency ^11^. This may explain how patients with Barlow’s disease can have a regurgitation severity that is quantitatively similar to that of patients with fibroelastic deficiency despite having greater prolapse severity and annular enlargement. Patients with Barlow’s disease are able to compensate through increased mitral tissue distensibility during mid to late systole ^11^. Patients with fibroelastic deficiency have reduced systolic leaflet area changes, indicating little tissue reserve. Thus, despite relatively preserved annular function, they develop severe regurgitation with “fewer” morphological changes.

Overall, there are important surgical and percutaneous implications if fibroelastic deficiency and Barlow’s disease are distinct entities rather than morphological variants ^16^. These results suggest that an understanding of the pathophysiological changes is needed in planning surgical repair. This is so that different surgical approaches may be used to achieve a successful and durable repair. For instance, resection of excessive leaflet tissue may have a different impact in a patient with Barlow’s disease where regurgitation is reduced by leaflet distensibility versus a patient with fibroelastic deficiency where regurgitation is increased because of a lack of leaflet reserve. Similarly, the success of surgical or percutaneous edge-to-edge mitral repair methods may depend on the underlying pathophysiological changes.

Cardiac magnetic resonance imaging has been demonstrated to offer mechanistic insights into the interaction between the myocardium and myxomatous mitral regurgitation through the use of delayed or late gadolinium enhancement imaging and native/pre-contrast or post-contrast T _1_ mapping ^17^. Papillary muscle fibrosis with extension to the infero-basal wall, identified with late gadolinium enhancement imaging, has been observed in patients with mitral valve prolapse and a history of ventricular arrhythmias or sudden death ^17, 18^. This is likely due to excessive systolic leaflet motion resulting in mechanical stretch of the infero-basal papillary muscle and wall with resultant hypertrophy and scarring. Interestingly, patients did not necessarily have significant mitral regurgitation to develop this fibrosis and even a small burden of late gadolinium enhancement was associated with sudden cardiac death. It may be that late gadolinium enhancement does not identify all of the myocardial abnormalities that are present. Studies characterizing myocardial tissue with cardiac magnetic resonance have described the presence of diffuse interstitial derangement even in the absence of focal fibrosis and have shown that these changes are linked to subclinical systolic left ventricular dysfunction and ventricular arrhythmias in mitral valve prolapse with regurgitation ^19, 20^. Overall, these results suggest that arrhythmic mitral valve prolapse requires management that is different from that of echocardiographic mitral valve prolapse. Features that may identify these patients include mitral valve prolapse patients with mitral annular disjunction and systolic curling of the posterior leaflet, which are associated with a relative increase in basal to mid left ventricular hypertrophy and fibrosis ^21, 22^. However, these features may be late markers of myocardial changes. Given the large number of patients with mitral valve prolapse with or without significant regurgitation, systematic routine screening with cardiac magnetic resonance imaging is not feasible or even reasonable at this time. Additional studies are needed to determine who would benefit from arrhythmic risk stratification and which clinical, imaging or even genetic markers should be used.

In addition to developments in understanding pathomorphological mitral valve changes, an increase in appreciation of the presence and frequency of mitral valve clefts and cleft-like indentations has taken place in the past few years ^23, 24^. This is due in part to the increasing use of three-dimensional echocardiographic *en face* imaging, which allows complete visualization of the mitral valve in a single image. It is also due to the increasing number of patients undergoing assessment and treatment with mitral clip. Whereas mitral valve clefts have long been recognized as a source of residual regurgitation during surgical repair, the role of cleft-like indentations causing regurgitation post-mitral clip placement is now being recognized ^25^. Clipping can open up a cleft-like indentation with subsequent regurgitation, making it difficult to determine the likelihood of reducing mitral regurgitation severity post-clip.

## Epidemiology of mitral regurgitation

There is great interest in defining the number of patients with significant mitral regurgitation who are undertreated because of the resources that are being devoted to developing costly new therapeutic interventions for mitral regurgitation. Studies have reported that up to 50% of patients with moderate to severe mitral regurgitation who are referred for surgical evaluation are denied intervention ^26^. The number of undertreated patients is likely higher when those who have not been referred for intervention are included and this is not accounting for the substantial number of patients with significant mitral regurgitation who are undiagnosed ^27– 29^. One clue to the size of this undertreated population comes from the known gap between the number of patients in epidemiological studies with significant mitral regurgitation and the number of isolated mitral valve surgeries performed yearly ^30, 31^. Supporting this idea that mitral valve operations are likely underperformed is the fact that isolated and combined aortic valve surgeries (excluding transcatheter procedures) are performed 1.6 times more commonly than mitral valve operations despite a two- to three-fold higher prevalence of mitral valve disease ^31^.

However, there are many reasons why patients may be denied intervention. These include socioeconomic factors such as poor access to care and treatment and clinical factors related to late referral for intervention and the presence of co-morbidities precluding intervention. It was unclear whether the number who would be considered undertreated would be so large if issues relating to access to care and treatment could be removed. Dziadzko *et al*. addressed this knowledge gap by studying a population with easy access to diagnostic and therapeutic interventions, which allowed them to separate socioeconomic from clinical factors ^32^. They examined a group of patients whose isolated moderate or severe mitral regurgitation was diagnosed between 2000 and 2010 in Olmsted County (Minnesota, USA), the main catchment region for the Mayo Clinic ^32^. They identified 1294 patients (0.6% of all assessed adults) with moderate or severe mitral regurgitation, of whom 538 (42%) had a left ventricular ejection fraction below 50%. Overall, these patients with moderate or severe mitral regurgitation had higher rates of mortality compared with the general population in that region and this was true even if they had few co-morbidities and normal left ventricular function. Not surprisingly, those with poor left ventricular function had poorer outcomes. Despite access to the resources of the Mayo Clinic, only 15% of the 1294 patients underwent surgical therapy. Even within the subgroup of 561 patients with a left ventricular ejection fraction of at least 50% and class 1 guideline-based indications for intervention, only 118 patients underwent surgery. Even more concerning was the finding that men, despite having more co-morbidities, were twice as likely than women to have mitral surgery.

While this article confirms that there is a large group of patients who are undertreated despite adequate access to care and intervention, it also suggests that there are areas in current clinical practice that could be improved before consigning all of these patients to percutaneous procedures. It is unclear why patients with significant mitral regurgitation are not undergoing intervention as per societal guidelines. Whether this is due to patient reluctance to undergo surgery, overestimation of the risks over the benefits of mitral valve surgery by the managing physicians, or reluctance of the surgeon to operate is unclear. Also, women are significantly undertreated. Although this is not a new finding, it warrants stronger actions to improve outcomes in women ^33, 34^. More data are needed to understand why it occurs. Perhaps societal guidelines require cutoff values indexed to body surface area to account for sex difference or more objective testing for symptoms should be instituted.

## Management

### Changes to the guidelines

Both the American Heart Association/American College of Cardiology ^1^ and the European Society of Cardiology ^3^ guidelines for managing patients with valvular heart disease were updated in 2017. Changes in these guidelines include grading of mitral regurgitation severity in primary and secondary mitral regurgitation, the use of valve replacement over repair in severe secondary mitral regurgitation, treatment of moderate secondary mitral regurgitation at the time of coronary artery bypass surgery, and an inclination to earlier intervention in asymptomatic patients with severe primary mitral regurgitation ( Table 3).

**Table 3.** Mitral valve guideline changes.

Disease	American guideline changes	European guideline changes
Primary mitral regurgitation	New IIa recommendation for surgery in asymptomatic patients with severe primary mitral regurgitation, normal left ventricular (LV) size (LV end-systolic diameter <40 mm) and ejection fraction (>60%), with progressive LV enlargement or decline in ejection fraction on serial imaging. The patient must also be a low surgical risk and the chance of repair must be high.	New IIa recommendation for surgery for an asymptomatic patient with severe primary mitral regurgitation who is in sinus rhythm with an LV ejection fraction of more than 60% if the probability of repair is high, the LV end-systolic diameter is between 40 and 44 mm, and one of the following criteria is met: the left atrium is dilated (≥60 mL/m ^2^) or the cause is chordae tendineae rupture. Patients who have severe pulmonary hypertension with exercise are not included in this indication for surgery.
Secondary mitral regurgitation	Cutoff values for severe regurgitation are the same regardless of primary or secondary etiology • effective regurgitant orifice area ≥0.4 cm ^2^ • regurgitant volume ≥60 mL	No change
	New IIa recommendation for chordal-sparing mitral valve replacement over mitral valve repair in those with symptomatic severe secondary mitral regurgitation despite optimal medical therapy	European guidelines still lean toward restrictive annuloplasty in these patients.
	American guidelines reflect uncertainty on the role of mitral valve repair in patients with moderate mitral regurgitation at the time of bypass surgery.	European guidelines have removed the indication for valve intervention in patients with moderate mitral regurgitation at the time of bypass surgery.
		New IIb recommendation for percutaneous intervention in patients with severe ventricular dysfunction despite optimal medical therapy if surgical risk is high.

With respect to the mitral valve, the American guidelines were notable for unifying cutoff values for grading mitral regurgitation severity ^1^. The previous guidelines, published in 2014, had emphasized the need to differentiate between primary and secondary mitral regurgitation and recommended lower cutoffs for reporting severe regurgitation in patients with secondary mitral regurgitation ^5^. This recommendation was based on studies demonstrating greater adverse outcomes with smaller effective regurgitant orifice areas in patients with secondary mitral regurgitation. This change created difficulties in reporting mitral regurgitation severity because mitral regurgitation etiology is not always apparent in the echocardiographic laboratory and the patient’s clinical status (that is, in overt failure or clinically stable) is often unknown. Currently, for both primary and secondary mitral regurgitation, severe mitral regurgitation is defined as an effective regurgitant orifice area of at least 0.4 cm ^2^ or a regurgitant volume of at least 60 mL. Although this simplifies clinical reporting, the intent of the original recommendation should still be remembered. It was meant to raise awareness that patients with secondary mitral regurgitation may be classified under current guidelines as moderate but have a risk of adverse events similar to those classified as severe. However, intervention on the valve in these patients has not been demonstrated to improve outcomes.

For patients with secondary mitral regurgitation, the American guidelines included a new class IIa recommendation to choose chordal-sparing mitral valve replacement over annuloplasty with repair in those with chronic severe mitral regurgitation despite goal-directed medical therapy. This recommendation originates from the results of a randomized clinical trial on patients with secondary mitral regurgitation who were randomly assigned to mitral valve repair or replacement ^35, 36^. Although both groups demonstrated improvements in ventricular remodeling, the repair group has higher rates of recurrent moderate or severe mitral regurgitation, heart failure and repeat cardiac hospitalizations. However, patients with durable repairs had better outcomes than those with recurrent regurgitation. Also, recurrent regurgitation after annuloplasty was often due to tethering of the posterior leaflet toward the apex, which was not addressed by the annuloplasty ring. Thus, the 2017 European guidelines still lean toward recommending repair with a restrictive annuloplasty for these patients, as some patients do benefit from repair over replacement. What is unclear is how to identify these patients and what other repair techniques should be used in addition to the annuloplasty ring to improve repair durability.

For patients with moderate secondary mitral regurgitation undergoing coronary artery bypass surgery, the American guidelines reported uncertainty regarding whether this valve should be repaired at the time of surgery. This is due to a study that randomly assigned patients with moderate mitral regurgitation at the time of coronary artery bypass surgery to mitral valve repair plus bypass surgery or to bypass surgery alone ^37, 38^. The European guidelines have withdrawn the indication for mitral valve surgery during coronary bypass surgery.

For secondary mitral regurgitation, the European guidelines also included a new IIb indication for percutaneous intervention. Patients with severe ventricular dysfunction who have no indication for revascularization and are symptomatic despite optimal medical therapy may require surgery if the surgical risk is low or percutaneous intervention if the surgical risk is high and valve morphology is favorable. This recommendation depends on the patient’s left ventricular ejection fraction. For those with a left ventricular ejection fraction of not more than 30%, there is no evidence that treatment of secondary mitral regurgitation improves survival.

For primary regurgitation, the American guidelines include a new class IIa recommendation based on a level of evidence C-LD that mitral valve surgery is reasonable for asymptomatic patients with primary mitral regurgitation who have normal left ventricular size (left ventricular end-systolic diameter of less than 40 mm) and ejection fraction (>60%) and have progressive left ventricular enlargement or a reduction in left ventricular ejection fraction on serial imaging studies. The patient must also have low surgical risk and the chance of repair must be high. This is due to increasing evidence demonstrating improved outcomes in patients who undergo surgery prior to achieving traditional surgical triggers ^39, 40^. In contrast, the European guidelines introduced a IIa recommendation for surgery in asymptomatic patients with severe primary mitral regurgitation in sinus rhythm with a left ventricular ejection fraction of more than 60% if the probability of repair is high, the left ventricular end-systolic diameter is between 40 and 44 mm, and one of the following criteria is met: the left atrium is dilated (≥60 mL/m ^2^) or the cause is chordae tendineae rupture. Patients who have severe pulmonary hypertension with exercise are not included in this indication for surgery. This differs from the American recommendation which emphasizes progressive changes on serial imaging and suggests that repair be performed only if it is feasible with a high rate of success and durability.

### Risk scores in mitral regurgitation

Although the guidelines offer direction for management, the decision for referral for intervention is an individual one based on patient co-morbidities, disease severity, and the likelihood of valve repair. Such decisions are often challenging and require a mental scale to subjectively weigh the value of various considerations. This leads to uncertainly and often a conservative management approach, which may be to the patient’s detriment. An example of this is the undertreatment of patients with significant mitral regurgitation from myxomatous mitral valve disease. Physicians tend to underestimate the mortality risks from the regurgitation and overestimate the risks from the patient’s co-morbidities ^41, 42^. The development of risk scores may help in these situations and two such scores have been developed in the past few years to improve management of patients with mitral regurgitation from myxomatous mitral valve disease.

The Mitral Regurgitation International Database (MIDA) score is an integrated risk score that was developed for use at the time of diagnosis to help improve risk stratification in patients with severe regurgitation from myxomatous mitral valve disease ( Table 4) ^43^. It is composed of clinical and echocardiographic parameters that are easily obtainable and includes risk factors that are associated with adverse outcomes in patients with myxomatous mitral valve disease. The score is meant to be used to determine whether a patient should continue medical therapy or undergo surgical intervention. With higher scores, there was a progressive increase in 1- and 5-year mortality in patients who were medically managed and those who underwent surgery. Compared with the EuroSCORE II, the MIDA score provided incremental prognostic predictive value. Although the score was validated, it was in a population that differed only in that it included patients with prolapse in addition to flail leaflets. It must be noted that this score did not include measurements from three-dimensional echocardiography, global longitudinal strain, the presence of fibrosis on cardiac magnetic resonance imaging, peptides, or exercise testing, all of which could improve the MIDA score.

**Table 4.** Score for risk stratification with myxomatous mitral valve disease.

Factors	Points
Age ≥ 65 years	3
Symptoms	3
Right ventricular systolic pressure > 50 mm Hg	2
Atrial fibrillation	1
Left atrial diameter ≥ 55 mm	1
Left ventricular end-systolic diameter ≥40 mm	1
Left ventricular ejection fraction ≤ 60%	1

It is recognized that mitral valve repair is superior to replacement in patients with mitral regurgitation secondary to myxomatous mitral valve disease. Ideally, all patients would have a successful repair at the time of surgery that is durable and long-lasting. Unfortunately, in the real world, there is a spectrum of repair success rates and durability that is related to surgeon volume ^44^. Stratification of patients by mitral valve lesion complexity may assist in directing those with complex etiologies or lesions to surgeons with greater expertise and volumes and so improve repair rates. To date, determining complexity has been based on the knowledge that anterior leaflet repair is more difficult than posterior leaflet repair and multi-segmental posterior leaflet repair is more complicated than single segment repair. A scoring system for determining complexity in patients with myxomatous mitral valve disease has been proposed ( Table 5) ^45^. A score of 1 is considered a simple repair, 2 to 4 is considered intermediate and 5 or more is complex. This score has the potential to assist the cardiologist in determining the surgeon whom their patient should be referred to and the surgeon in planning and predicting reparability. Some limits to this study were the arbitrary manner in which weighting for the scores was assigned and the high repair rate at the center where this score was devised. Additionally, this score was not prospectively validated. However, such scoring is needed to help guide management of these patients as a high proportion of patients unfortunately are not appropriately receiving valve repair. It also simplifies the decision making when compared with the table in the 2017 Expert Consensus paper published by the American College of Cardiology, which classifies the feasibility of repair as ideal, challenging or contraindicated without recognizing the spectrum and combination of abnormalities that may be present ( Table 6) ^4^.

**Table 5.** Risk stratification of complexity for repair of myxomatous mitral valves.

Anatomic factors	Points
Segment prolapse	
P1	1
P2	1
P3	1
A1	2
A2	2
A3	2
Anterolateral commissure prolapse	2
Posteromedial commissure prolapse	2
Any leaflet restriction	2
Papillary muscle or leaflet calcification without annular involvement	2
Annular calcification	3
Previous mitral valve repair	3

**Table 6.** Feasibility of mitral valve repair from the American College of Cardiology 2017 Expert Consensus Decision Pathway on the Management of Mitral Regurgitation ^42^.

Parameter	Ideal pathoanatomy	Challenging pathoanatomy	Contraindicated pathoanatomy
Primary lesion location	Posterior leaflet only	Anterior leaflet or bileaflet	None
Leaflet calcification	None	Mild	Moderate to severe
Annular calcification	None	Mild to moderate with minimal leaflet encroachment	Severe or with significant leaflet encroachment
Subvalvular apparatus	Thin, normal	Mild diffuse thickening or moderate focal thickening	Severe and diffuse thickening with leaflet retraction
Mechanism of mitral regurgitation	Type II fibroelastic deficiency or focal myxomatous prolapse or flail	Type II forme fruste or bileaflet myxomatous disease; type I healed or active endocarditis, type IIIA/B with mild restriction or leaflet thickening	Type IIIB with severe tethering and infero-basal aneurysm; type IIIA with severe bileaflet calcification; type I active infection with severe leaflet or annular tissue destruction
Unique anatomic complexities	None	Redo cardiac operation or mitral re-repair; anatomic predictors of systolic anterior motion, adult congenital anomalies; focal papillary muscle rupture	Mitral valve reoperation with paucity of leaflet tissue; diffuse radiation valvulopathy; papillary muscle rupture with shock

### Impact from choosing a bioprosthetic mitral valve

Between 60% and 70% of patients who undergo mitral valve replacement surgery receive a bioprosthetic valve and this proportion is growing ^31, 46^. However, there are many uncertainties that persist with the use of bioprosthetic mitral valve. For patients undergoing prosthetic mitral valve replacement surgery, the decision regarding the choice of prosthesis—mechanical or bioprosthetic—is generally based on a discussion concerning the need for anticoagulation with mechanical valves, the belief of improved durability with current bioprostheses, and patient risks for reoperation. This discussion assumes that mortality rates of patients who receive a bioprosthetic valve are equal to those of patients who receive a mechanical valve ^47, 48^. However, there is growing evidence, especially for patients under the age of 70 years, that long-term all-cause mortality is higher in patients with a bioprosthetic valve in the mitral position ^46, 49^. One large study, based on administrative data, reported that patients between the ages of 40 to 69 years who receive a bioprosthetic mitral valve have significantly higher 15-year mortality rates compared with those who received a mechanical mitral valve ^46^. Critics of this study have pointed out that there may have been co-morbidities affecting life expectancy in those who receive a bioprosthetic mitral valve which in turn affect outcomes. Also, changes in the bioprosthetic valve technology affecting durability during the study period may have accounted for some of the adverse outcomes observed in the study. Finally, many cardiologists believe that should the bioprosthetic mitral valve fail, percutaneous mitral valve replacement could be performed and so decision making should account for this management option. This may be an important option when the patient is young and possibly facing many sternotomies. Whether this is a reasonable strategy needs further discussion, as will be discussed in the next section.

### Surgical versus transcatheter mitral valve-in-valve/ring

**Figure 2.**
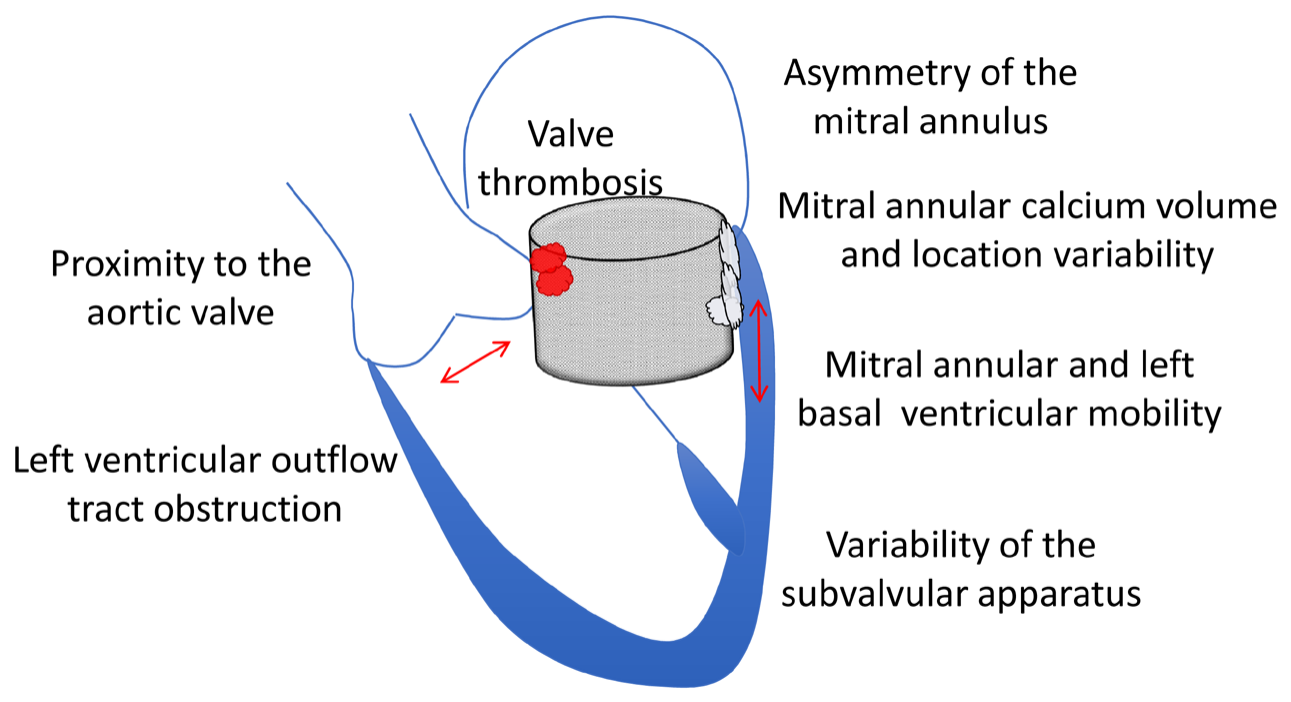
Some of the challenges of transcatheter mitral valve development.

All bioprosthetic valves degenerate, affecting short- and long-term patient outcomes. Up to one third of patients may require redo mitral valve surgery after a median of 8 years, and the rate of degeneration is faster in younger patients ^46, 47, 50^. Although individual risks will vary, mortality rates in the published literature for reoperation on the mitral valve range from 8% to 14% ^46, 50– 52^. It is knowledge of these high risks during reoperation that is leading providers to refer patients for transcatheter options and manage patients with the belief of potential transcatheter intervention ^53^. However, a series of publications in the last few years have suggested that redo mitral valve surgery is being increasingly performed, and with more surgical experience, the risks of redo mitral surgery have decreased dramatically ^50, 51, 54^. Additionally, for carefully selected patients, who do not have endocarditis, heart failure, or the need for a concomitant procedure, mortality can be as low as 4.1% to 5.5% ^52, 55^. Although the risks of reoperation are still higher than the risks during a first operation, continued reduction in redo surgical risks will affect the standards that transcatheter valve-in-valve replacements will need to meet for widespread clinical use. This is especially true given that long-term durability of catheter-based valves in the mitral position is unknown, and at least one study has demonstrated higher mitral valve gradients at 1 year compared with a surgical redo mitral valve replacement ^56^. Complications from transcatheter mitral valve-in-valve procedures are also high, emphasizing the need for careful patient selection. In one study, 73 complications ranging from left ventricular outflow tract obstruction, left ventricular perforation and post-procedural mitral stenosis, mitral valve thrombosis, and residual mitral valvular and paravalvular regurgitation occurred in 87 patients ^57^. Another interesting point seen in these studies is that despite the enthusiasm for valve-in-valve/ring procedures, the number of patients who could be considered for a transcatheter mitral valve-in-valve or valve-in-ring procedure may not be large. One study identified 121 patients at three sites over 10 years ^56^. Another found 147 patients in 24 years (or six patients a year); of these patients, only 36 had a Society of Thoracic Surgeons predictors-of-mortality score greater than 4%, further limiting the number of potential candidates ^55^. This number may change as fewer mechanical valves and annuloplasty bands are implanted to allow possible percutaneous intervention after an initial surgery ^58^. Overall, although there may be an understandable preference for mitral valve-in-valve procedures, there are many unknowns prior to wider use and extension into native valve in healthier patients ( Figure 2).

### Transcatheter mitral valve repair

Although there have been and continue to be many transcatheter mitral valve repair techniques in development, only edge-to-edge repair through the MitraClip procedure has become widely adopted in routine clinical practice ^59, 60^. It is typically performed in patients with symptomatic regurgitation who have limited treatment options as the procedure has good success at reducing regurgitation and low procedural mortality ^61, 62^. With greater use, indications have expanded with its use in anatomically challenging cases as well as patients with lower surgical risks ^60^. However, data on the MitraClip beyond 5 years are limited. The EVEREST II trial that compared MitraClip with surgery found that MitraClip at 5 years was inferior to surgery because of recurrent regurgitation. If this occurs in clinical practice, it would indicate that durability of results is limited and patients who are selected for this procedure should be chosen with this eventual result in mind. With respect to the other transcatheter techniques, it would be ideal to be able to offer combined techniques such as transcatheter annuloplasty and leaflet intervention to achieve complete transcatheter repair of the valve across the range of mitral valve pathologies and perhaps improve durability. However, this has yet to come to fruition.

There has been great anticipation for the publication of two trials—MITRA-FR and COAPT—which have studied the utility of MitraClip in patients with symptomatic severe functional mitral regurgitation ^63, 64^. These two trials had very different results. MITRA-FR randomly assigned 304 patients to either optimal medical therapy or MitraClip and found no difference in rehospitalization or all-cause death at 1 year. In contrast, COAPT randomly assigned 610 patients to either guideline-directed medical care or guideline-directed medical care plus MitraClip implantation and found that all hospitalizations and mortality at 24 months were lower in the MitraClip arm. There are differences between these two trials that may explain some of the differences. In COAPT, patients were highly selected as they had to be on maximally tolerated medical therapy prior to enrollment in the trial. The stricter criteria for enrollment in COAPT resulted in a slower rate of enrollment in the trial, which required 8 years to complete. Enrollment in MITRA-FR did not have any medication requirements, but it was a pragmatic trial studying the types of patients who are currently referred for MitraClip. Patients in COAPT had more severe mitral regurgitation with a mean effective orifice area of 41 mm ^2^ compared with 31 mm ^2^ in MITRA-FR, but these patients also had smaller left ventricular end-diastolic volumes (101 mL/m ^2^ in COAPT versus 135 mL/m ^2^ in MITRA-FR), suggesting that their disease may not have been as severe. However, mortality rates in the control arms for the two trials were similar. Lastly, residual mitral regurgitation was higher in the MITRA-FR group (17%) than in COAPT (5%). Overall, these results suggest that mitral regurgitation not only is a marker of heart failure severity but contributes to the abnormal pathophysiology in patients with heart failure. The results also suggest that there may be a population of patients with symptomatic severe secondary mitral regurgitation that will benefit from intervention. It has been postulated that this population has disproportionate secondary mitral regurgitation, where the quantified mitral regurgitation is out of keeping with the size of the left ventricle ^65^. However, more study to better define this population echocardiographically is needed. What is also not known from these studies is what happened to the brain natriuretic protein levels in the COAPT group and how novel therapies such as sacubitril/valsartan would have affected the results of the COAPT trial. Ultimately, the results of a third trial in this population, the RESHAPE HF2 trial, may bring more clarity to this area.

Beyond MitraClip, other devices have been developed for percutaneous edge-to-edge repair. This includes the Edwards PASCAL device that permits better deployment with simpler left atrial navigation and independent leaflet grasping ^66^. It also has a central spacer that provides addition mitral regurgitation reduction. A feasibility study in 23 patients has been published, and a multi-center trial of 120 patients is under way ^67^. Outside of the edge-to-edge devices, there are percutaneous chordal replacement devices and devices that address mitral annular enlargement, such as coronary sinus annuloplasty, and incomplete and complete annuloplasty rings. Percutaneous chordal replacement devices are growing in popularity and several large studies are under way ^68^. Percutaneous mitral annuloplasty devices have been implanted mainly in patients with functional or secondary mitral regurgitation and fewer than 1500 patients worldwide have received these devices compared with the 60,000 who have received the MitraClip ^67^. Ultimately, the future may become one where combinations of these percutaneous mitral valve repair devices are used, mimicking current surgical repair techniques ^69, 70^. However, the costs of these devices individually are quite high and so combining their use would require either that the prices decrease or that significant benefits be demonstrated in clinical trials with very broad populations.

### Transcatheter mitral valve replacement

Beyond developing transcatheter mitral valve repair, investigators are developing novel methods for transcatheter mitral valve replacement (TMVR) ^71^. Compared with transcatheter aortic valve replacement, TMVR has been more challenging because of the size and anatomy of the mitral valve, the lack of calcium to act as anchors in patients with mitral regurgitation, and the risk of left ventricular outflow tract obstruction ^72^. Additionally, there have been challenges in recruiting patients for these trials; rejection rates have been about 60% to 70% ^73, 74^. Of those excluded, about 20% to 50% of patients are excluded because of anatomic concerns and 20% to 40% for clinical or other issues ^75^. Overall, patients are being carefully selected to avoid intervention failure due to treatment of those with disease to advance to improve or those who are at risk of complications due to anatomic features. But the highly selective nature of these studies raises concerns regarding the real-life applicability of these devices.

Recent developments hold the promise of increasing the population that could be treated. One is the development of a transseptal approach ^74^. Although only 10 patients were studied with this approach, procedural success was high and safe. This is encouraging but it must be noted that this less invasive approach does have some limitations related to intracardiac maneuverability and sheath sizes ^75^. With respect to anatomic features, the two most common reasons for exclusion have been left ventricular dimensions that are out of the treatable size of the device under study and possible post-procedural left ventricular outflow tract obstruction ^75^. This latter issue was addressed with a novice device that lacerates the anterior leaflet prior to TMVR implantation ^76^. In 30 patients, this approach was found to be safe and effective in preventing left ventricular outflow tract obstruction. However, these patients still experienced issues related to the TMVR device used. In general, TMVR is still in the very early stages of clinical use. More study will be needed with respect to patient selection, the duration of anticoagulation required, the timing and mode of valve degeneration, and the impact of left ventricular remodeling after intervention on device stability.

### Mitral stenosis

The publications on treating and managing mitral stenosis have not been as plentiful as those on mitral regurgitation and this is reflected by a lack of changes in the guidelines with respect to managing mitral stenosis. One area that is developing is the growing experience with mitral valve repair in rheumatic mitral valve disease. Literature in this field should be interpreted with the recognition that there are differences between patients with rheumatic mitral valve disease who undergo surgery in developed countries compared with less developed nations ^77^. Patients in developed nations tend to be older at the time of valve surgery and have quiescent rheumatic disease, whereas those in developing nations tend to be young with active progressive or recurrent rheumatic disease, which would require reoperation. Additionally, differences in outcomes between mitral valve repair and replacement in developed countries are minimal because of the resources available to comply with mechanical replacement. Regardless of these differences, it has been suggested that both groups would benefit from mitral valve repair ^77^. In fact, another study demonstrated no difference in late mortality or the need for reoperation, and the risk of valve-related complication was significantly lower in the repair group ^78^. This is despite the younger average age in the repair group who also predominantly had regurgitation and the fact that there were very few repair cases performed per year per surgeon. This is consistent with other articles reporting that durability of mitral valve repair for rheumatic disease in the current era has improved and is comparable to the outstanding durability of repairs for degenerative disease ^79, 80^. Although much effort has been directed toward increasing mitral valve repair with degenerative mitral valve disease, perhaps the direction should be toward an expectation of valve repair if possible for all patients.

## Summary

Currently, there are many developments in the field of mitral valve disease that offer promise in improving our understanding and management of these patients. Application of novel imaging methods is providing us greater insights into mitral valve remodeling with disease and how these could contribute to possible mechanisms for failure with valve interventions. Additionally, although there is a significant number of patients with clinically important mitral regurgitation who are undertreated, advances in interventional techniques may not necessarily be required to improve their outcomes. Greater application of the guidelines and use of risk scores may have a larger impact. Also, the promise of transcatheter procedures will increase the number of patients receiving a bioprosthetic valve, but a clear discussion should be had with patients with respect to risks of mortality, failure and reoperation. Lastly, more study is needed both in transcatheter mitral valve procedures and in surgical repair to improve long-term patient outcomes.
